# Supplementary material for: In vivo spontaneous Ca2+ activity in the pre-hearing mammalian cochlea
Source: Nat Commun. 2025 Jan 2;16:29. doi: 10.1038/s41467-024-55519-w (PMC11695946; doi:10.1038/s41467-024-55519-w)
Supplement: Supplementary file 1 — Supplementary Information [file 41467_2024_55519_MOESM1_ESM.pdf]

***In vivo* spontaneous Ca^{2+} activity in the pre-hearing mammalian cochlea**

Francesca De Faveri, Federico Ceriani, Walter Marcotti

<u>This document includes:</u>

Supplementary Figures 1-9

Supplementary Figure 1

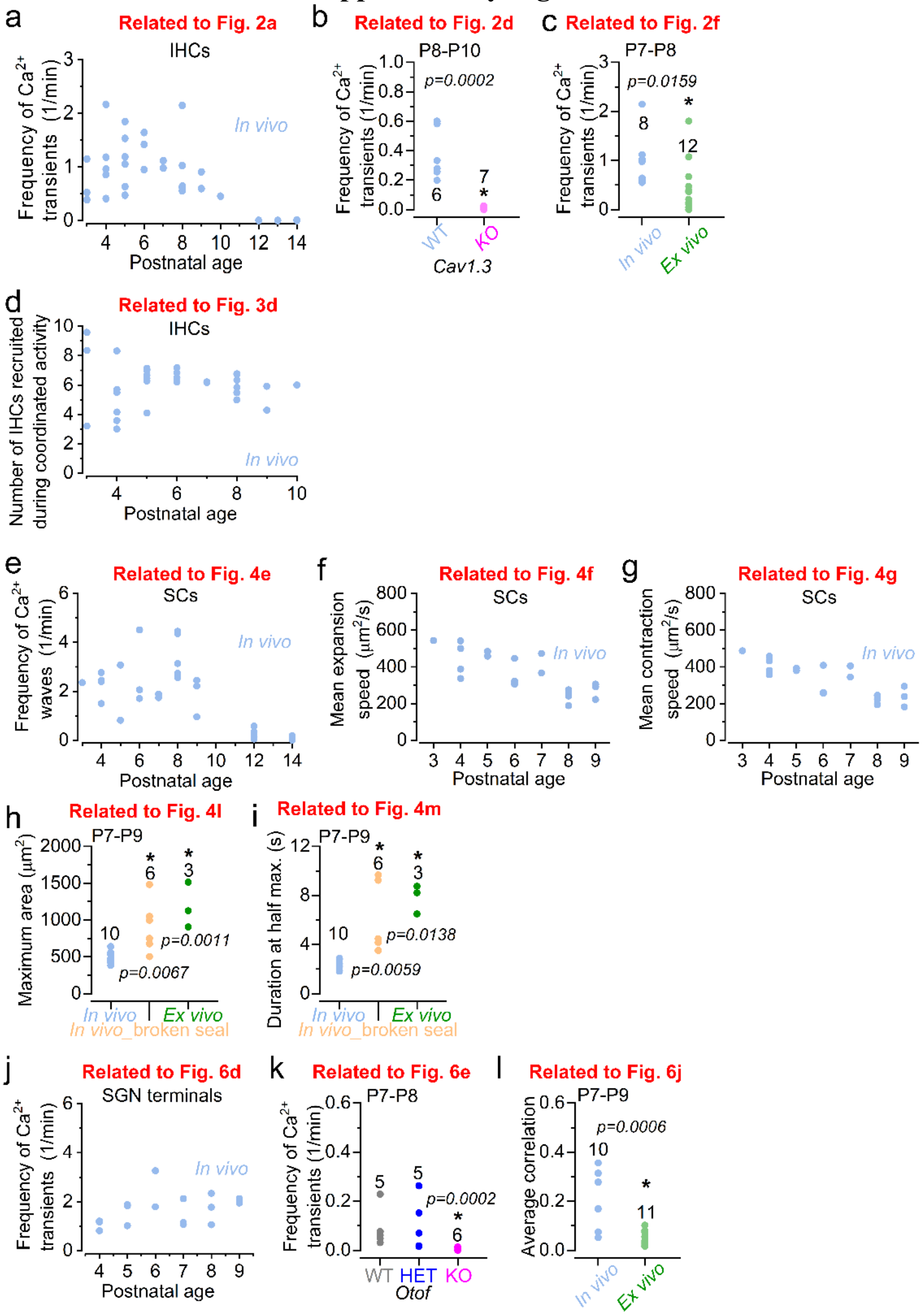

Supplementary Figure 1: Data plotted as average per mouse.

a-c, Data showing some features of Ca^{2+} transients in the inner hair cells (IHCs) from Fig. 2a (**a**), Fig. 2d (**b**) and Fig. 2f (**c**) in the main manuscript but plotted as averages per mouse. Statistical analysis: (**a**) age-relate changes in the frequency of Ca^{2+} transients *in vivo*, P=0.0375, Kruskal-Wallis test (number of mice from left to right: 3,5,6,3,2,6,2,1,2,1,3); (**b**) comparison of Ca^{2+} transient frequency between wild-type (WT, blue) and *Ca$_V$1.3* knockout (KO, magenta) mice, P=0.0002, Student's *t*-test; (**c**) comparison of Ca^{2+} transient frequency between *in vivo* (blue) and *ex vivo* (cochlear explants, green) recordings, P=0.0159, Mann Whitney *U*-test. Numbers above the data points in panels (**b**,**c**) represent the number of mice.

d, Data from Fig. 3d in the main manuscript but plotted as averages per mouse. Statistical analysis: age-related changes in the number of IHCs recruited during coordinated activity, P=0.3110, Kruskal-Wallis test. Number of mice from left to right: 3,6,7,4,2,6,2,1.

e-i, Data describing some features of Ca^{2+} waves in supporting cells (SCs) from Fig. 4e (**e**), Fig. 4f (**f**), Fig. 4g (**g**), Fig. 4l (**h**) and Fig. 4m (**i**) in the main manuscript but plotted as averages per mouse. Statistical analysis: (**e**) age-related changes in the frequency of Ca^{2+} waves, P=0.0009, Kruskal-Wallis test (number of mice from left to right: 1,4,2,3,2,5,3,6,7); (**f**) age-related changes in the mean expansion speed of Ca^{2+} waves, P=0.0136, Kruskal-Wallis test (number of mice from left to right: 1,4,2,3,2,5,3); (**g**) age-related changes in the mean contraction speed of Ca^{2+} waves, P=0.0236, Kruskal-Wallis test (number of mice from left to right: 1,4,2,3,2,5,3); Maximum area (**h**) and duration of half maximum (**i**) of Ca^{2+} waves from *in vivo* (blue), *in vivo* but with the broken membrane sealing the cochlear compartment (orange) and *ex vivo* (green) recordings, P=0.0011 and P=0.038, respectively, one-way ANOVA. Numbers above the data points in panels (**h**,**i**) represent the number of mice.
j-l, Data describing some of the features of Ca^{2+} transients in the spiral ganglion neuron (SGN) terminals from Fig. 6d (**j**), Fig. 6e (**k**) and Fig. 6j (**l**) in the main manuscript but plotted as average per mouse. Statistical analysis: (**j**) age-related changes in the frequency of Ca^{2+} transients, P=0.2494, Kruskal-Wallis test (number of mice from left to right: 4,3,2,4,4,3); (**k**) comparison of Ca^{2+} transient frequency between wild-type (WT), heterozygous (HET) and knockout (KO) mice for otoferlin (*Otof*), P=0.0002, Kruskal-Wallis test; (**l**) comparison of the average correlation of the Ca^{2+} transients between *in vivo* (blue) and *ex vivo* (green) recordings, P=0.0006, Student's *t*-test. Numbers above the data points in panels (**k,l**) represent the number of mice.
Data were tested for normality before performing statistical tests. Average data is included in the Source Data file.

Supplementary Figure 2

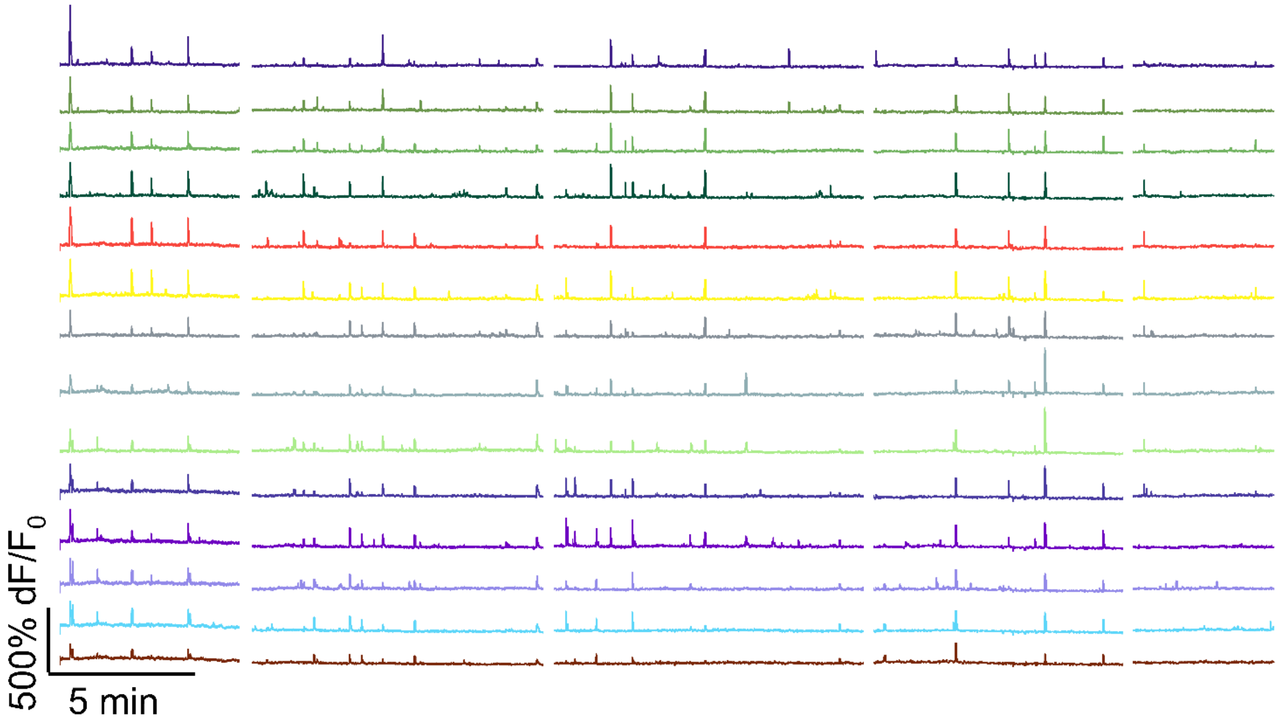

Supplementary Figure 2: Calcium transients in IHCs

Fluorescence time series highlighting spontaneous activity from individual IHCs *in vivo* in a representative timelapse recording (P3 *GCaMP6f*$^{fl/fl}$*Atoh1-cre*$^{+/-}$ mouse). Traces of the same colour represent subsequent recordings from the same IHC. The recording was stable for its entire duration (40.1 minutes of total recording time), with no discernible change in the fluorescence signal due to photobleaching and in the patterning of spontaneous Ca^{2+} transients. The four gaps in the traces indicate the stopping of the recording to manually reset the focal plane to its optimal position due to small drifts over time. For details on how these separate traces were processed, see Methods.

Supplementary Figure 3

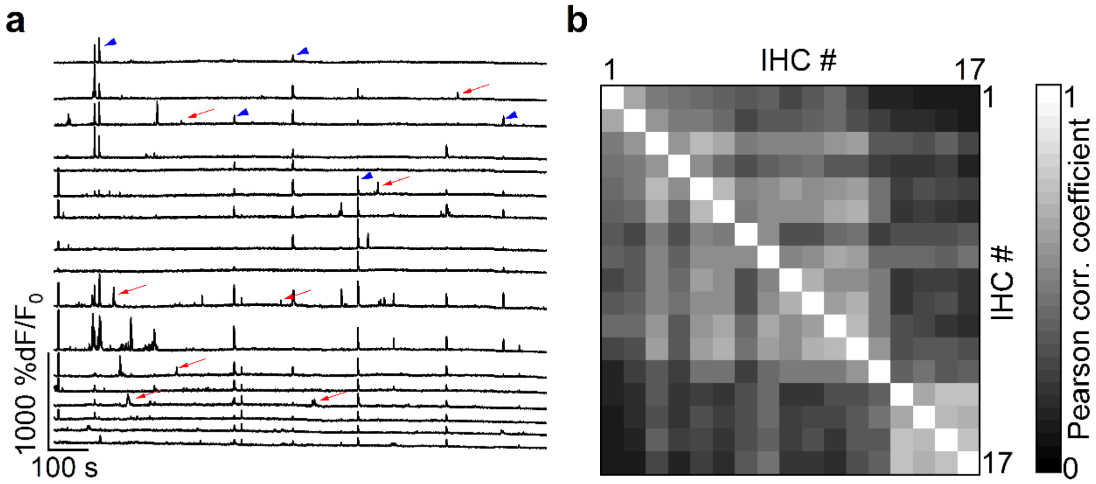

Supplementary Figure 3: Degree of correlated activity among adjacent IHCs.
a, Fluorescence traces from individual IHCs from a P3 *GCaMP6f$^{fl/fl}$Atoh-Cre$^{+/-}$* mouse, highlighting examples of spontaneous activity with a large number of single Ca^{2+} transients (red arrows) and some with coordinated activity (blue arrowheads). Traces represent adjacent IHCs. **b**, Correlation matrix for IHCs in panel **a**.

Supplementary Figure 4

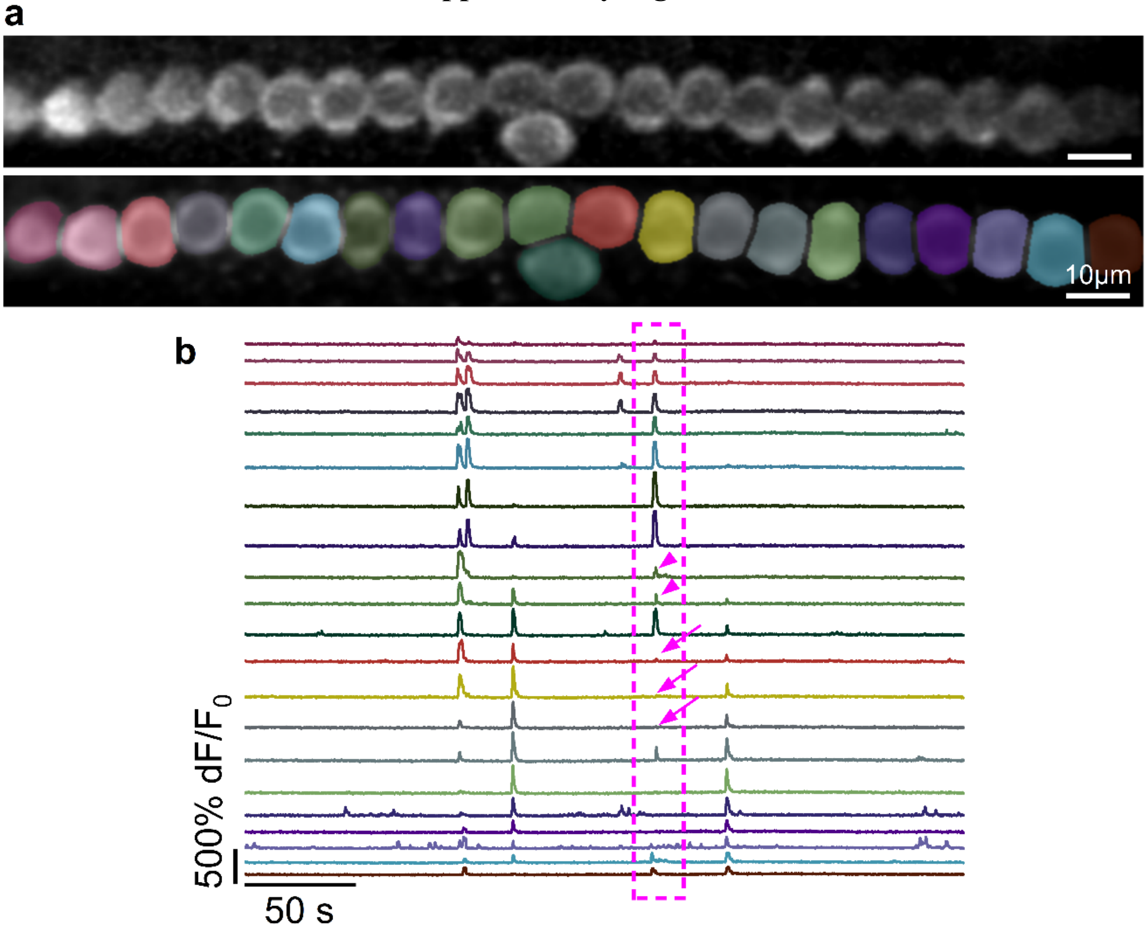

Supplementary Figure 4: Skipping Ca^{2+} transient in IHCs during coordinated activity

a, Top: average intensity projection of a timelapse recording highlighting GCaMP6f expression in the IHCs from the apical coil of a P5 *GCaMP6f$^{fl/fl}$Myo15-Cre$^{+/-}$* mouse. Bottom: ROIs generated using a semi-automated identification approach (see Methods), which were used to measure spontaneous Ca^{2+} signals from individual IHCs. **b**, Fluorescence time series computed as pixel-averages from the ROIs in **a**, highlighting spontaneous Ca^{2+} activity in IHCs (colour coded). Dashed magenta box indicates coordinated activity across several IHCs. Arrowheads: highly reduced Ca^{2+} transients; arrows: IHCs failing to elicit Ca^{2+} transients (skipping cells).

Supplementary Figure 5

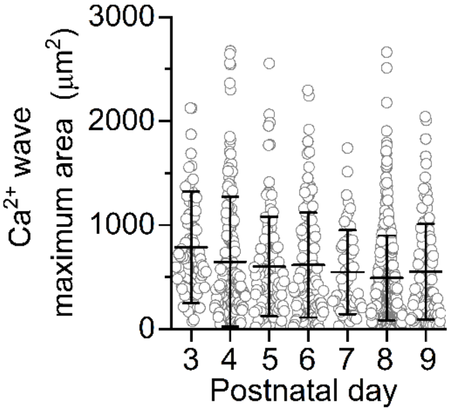

Supplementary Figure 5: Maximum area of Ca^{2+} wave propagation in supporting cells of the developing cochlea.

Maximum area of the Ca^{2+} waves. Number of waves from left to right: 96, 200, 113, 129, 54, 451, 116 from 20 mice. Average data are shown as mean ± SD.

Supplementary Figure 6

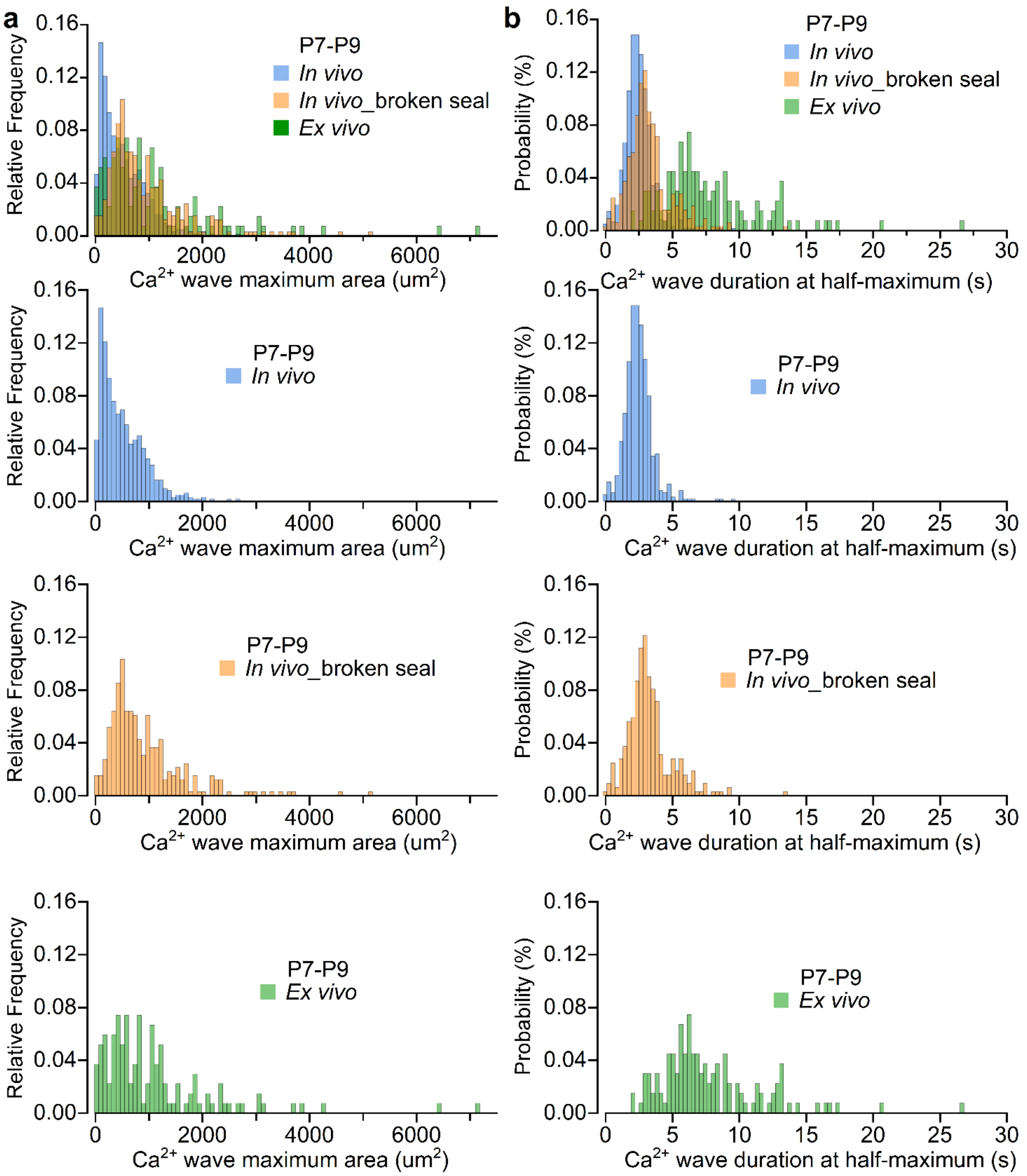

Supplementary Figure 6: Comparison of Ca^{2+} waves recorded *in vivo* and under unphysiological conditions.

a,**b**, Histograms showing the maximum area (**a**) and the full duration at half-maximum of the Ca^{2+} waves (**b**) for the combined (top panels) or separate (bottom 3 panels) three different experimental conditions: *in vivo* (blue), *in vivo* but with the rupture of the membrane sealing the cochlear partitions (orange) and from *ex vivo* preparations (green).

Supplementary Figure 7

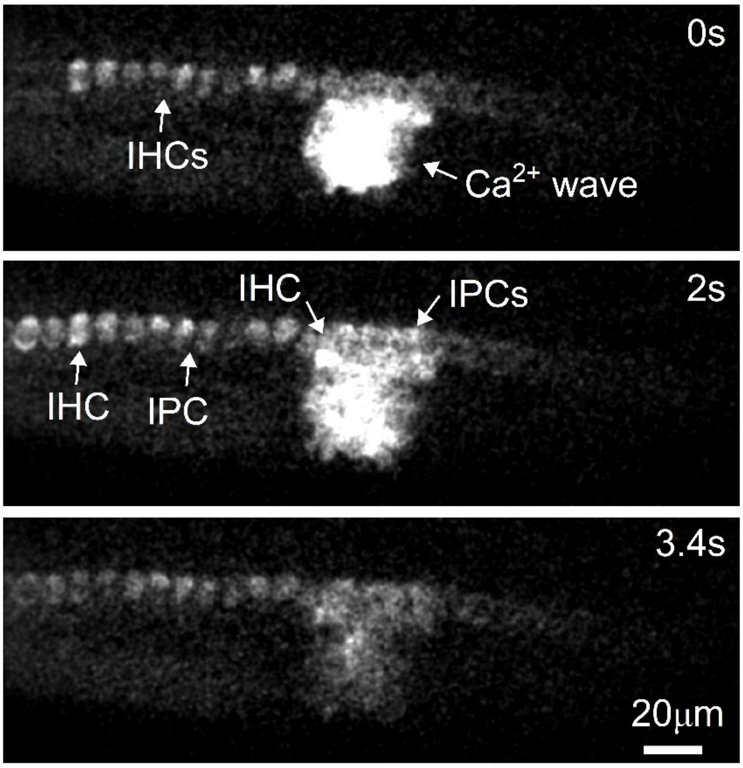

Supplementary Figure 7: Propagation of Ca^{2+} waves in supporting cells and IHCs.
Images from a recording from a P5 *GCaMP6f*$^{fl/fl}$*Pax2-Cre*$^{+/-}$ mouse showing the propagation of a spontaneous Ca^{2+} wave from the GER to IHCs. Each image is an average of 10 frames of the original timelapse recording. Notice the long-range propagation of the spontaneous activity in the IHC area. Inner phalangeal cells (IPCs) in between IHCs did not display Ca^{2+} elevations outside the area covered by the Ca^{2+} wave in the supporting cells of the GER.

Supplementary Figure 8

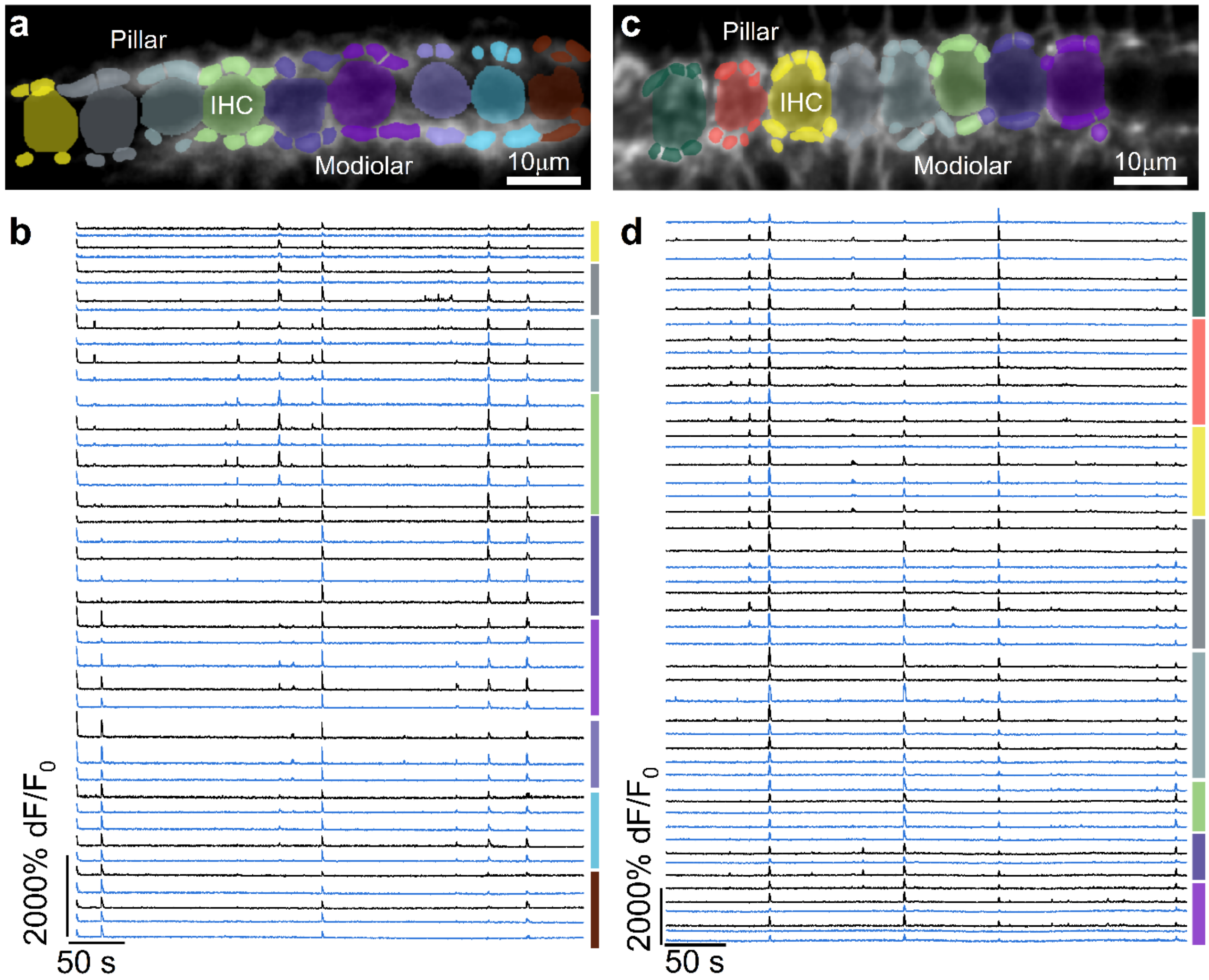

Supplementary Figure 8: Activity in individual postsynaptic terminals during single and coordinated activity across IHCs.

a,c, Average intensity projections displaying GCaMP6f expression *in vivo* from *GCaMP6f*$^{fl/fl}$*NeuroD-Cre*$^{+/-}$ mice (**a**, P4 mouse, **c**, P5 mouse), with superimposed segmentation mask highlighting ROIs for identified synaptic terminals (boutons) colour-matched to their associated IHC body. **b,d**, Fluorescence traces of pillar (blue) and modiolar (black) SGN terminals identified by the ROIs in panels **a,c** respectively. Colour labels on the right of the traces indicate the terminals belonging to the colour-matched IHC in panels **a,c**. Note that coordinated Ca^{2+} events across multiple IHCs consistently recruit a large number of boutons, while single IHC Ca^{2+} events show scattered activity in individual boutons.

Supplementary Figure 9

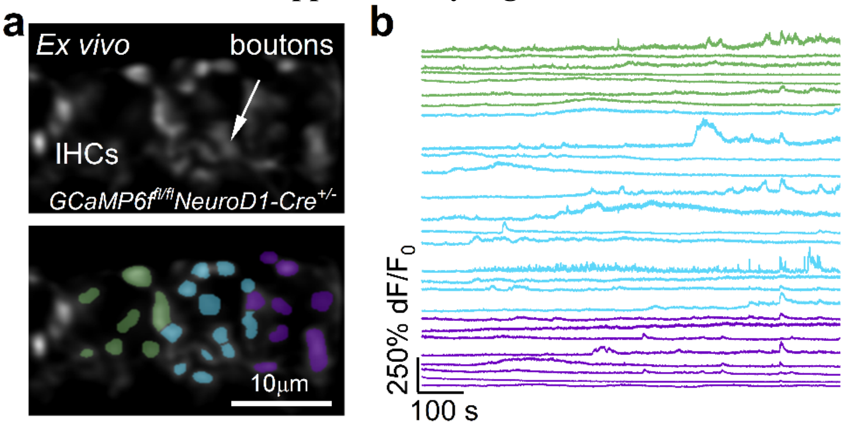

Supplementary Figure 9: Calcium activity in individual SGN terminals from explanted cochlea. **a**, Average intensity projection displaying GCaMP6 expression in a cochlear explant from a P8 mouse (*GCaMP6f*$^{fl/fl}$*NeuroD-Cre*$^{+/-}$) and segmentation mask highlighting ROIs (bottom) for identified synaptic boutons colour-matched to their associated IHC. **b,** Fluorescence signals from individual ROIs in (**a**).
