# Supplementary material for: In vivo spontaneous Ca2+ activity in the pre-hearing mammalian cochlea
Source: Nat Commun. 2025 Jan 2;16:29. doi: 10.1038/s41467-024-55519-w (PMC11695946; doi:10.1038/s41467-024-55519-w)
Supplement: Supplementary file 3 — Description of Additional Supplementary Files [file 41467_2024_55519_MOESM3_ESM.pdf]

Description of Additional Supplementary Files

Supplementary Movie 1 -Timelapse movie of spontaneous activity in IHCs of a P6 GCaMP6ffl/flAtoh1-Cre+/- mouse. The movie is reproduced at 3x speed. The movie is related to Fig. 1h,i of the main text. Note that instances where the movie appears to "freeze" are due to the replacement of motion artefacts with still images. The montage presents both the pre- (top) and post-processed images (bottom). See Methods for detailed description of image analysis.

Supplementary Movie 2- Related to Fig. 3a of the main text. Timelapse movie of spontaneous coordinated activity in IHCs of a P8 GCaMP6ffl/flMyo-Cre+/- mouse. The movie is reproduced at 3x speed. Still image sequence in Fig. 3a starts at 221 s. Note that instances where the movie appears to "freeze" are due to the replacement of motion artefacts with still images.

Supplementary Movie 3 -Timelapse movie of spontaneous Ca2+ wave activity in the GER of a P4 GCaMP6ffl/flPax2- Cre+/- mouse. The movie is reproduced at 3x speed. The movie is related to Fig. 4b of the main text. Note that instances where the movie appears to "freeze" are due to the replacement of motion artefacts with still images. The montage presents both the pre- (top) and post-processed images (bottom). See Methods for detailed description of image analysis.

Supplementary Movie 4 -Timelapse movie of Ca2+ signals in individual supporting cells around the IHCs of a P14 GCaMP6ffl/flPax2-Cre+/- mouse. The movie is reproduced at 3x speed. The movie is related to Fig. 4e of the main text. Note that instances where the movie appears to "freeze" are due to the replacement of motion artefacts with still images.

Supplementary Movie 5- Timelapse movie of spontaneous Ca2+ wave activity in the GER of a P4 GCaMP6ffl/flPax2- Cre+/- mouse, highlighting the spread of spontaneous activity in the bulk of the GER and IHC area. The movie is reproduced at 3x speed. The movie is related to Fig. 5a of the main text. Note that instances where the movie appears to "freeze" are due to the replacement of motion artefacts with still images.

Supplementary Movie 6- Timelapse movie of spontaneous coordinated activity in neuronal terminals contacting IHCs in a P4 Snap25-GCaMP6s mouse. The movie is reproduced at 3x speed. The movie is related to Fig. 6a-c of the main text. Note that instances where the movie appears to "freeze" are due to the replacement of motion artefacts with still images. The montage presents both the pre- (top) and post-processed images (bottom). See Methods for detailed description of image analysis.

Supplementary Movie 7- Timelapse movie of spontaneous activity in neuronal terminals contacting IHCs in a P4 GCaMP6ffl/flNeuroD1-Cre mouse. The movie is reproduced at 3x speed. The movie is related to Fig. 7 in the main text. Note that instances where the movie appears to "freeze" are due to the replacement of motion artefacts with still images.
