# Supplementary material for: In vivo spontaneous Ca2+ activity in the pre-hearing mammalian cochlea
Source: Nat Commun. 2025 Jan 2;16:29. doi: 10.1038/s41467-024-55519-w (PMC11695946; doi:10.1038/s41467-024-55519-w)
Supplement: Supplementary file 11 — Reporting Summary [file 41467_2024_55519_MOESM11_ESM.pdf]

nature portfolio

Corresponding author(s): Prof Walter Marcotti

Last updated by author(s): Nov 15, 2024

Reporting Summary

Nature Portfolio wishes to improve the reproducibility of the work that we publish. This form provides structure for consistency and transparency in reporting. For further information on Nature Portfolio policies, see our Editorial Policies and the Editorial Policy Checklist.

Statistics

For all statistical analyses, confirm that the following items are present in the figure legend, table legend, main text, or Methods section.

n/a	Confirmed	
☐	☒	The exact sample size (*n*) for each experimental group/condition, given as a discrete number and unit of measurement
☐	☒	A statement on whether measurements were taken from distinct samples or whether the same sample was measured repeatedly
☐	☒	The statistical test(s) used AND whether they are one- or two-sided<br>*Only common tests should be described solely by name; describe more complex techniques in the Methods section.*
☒	☐	A description of all covariates tested
☐	☒	A description of any assumptions or corrections, such as tests of normality and adjustment for multiple comparisons
☐	☒	A full description of the statistical parameters including central tendency (e.g. means) or other basic estimates (e.g. regression coefficient) AND variation (e.g. standard deviation) or associated estimates of uncertainty (e.g. confidence intervals)
☒	☐	For null hypothesis testing, the test statistic (e.g. *F*, *t*, *r*) with confidence intervals, effect sizes, degrees of freedom and *P* value noted<br>*Give P values as exact values whenever suitable.*
☒	☐	For Bayesian analysis, information on the choice of priors and Markov chain Monte Carlo settings
☒	☐	For hierarchical and complex designs, identification of the appropriate level for tests and full reporting of outcomes
☐	☒	Estimates of effect sizes (e.g. Cohen's *d*, Pearson's *r*), indicating how they were calculated

Our web collection on statistics for biologists contains articles on many of the points above.

Software and code

Policy information about availability of computer code

Data collection	ThorImage (Thorlab Inc., USA).
Data analysis	ImageJ (NIH), Python modules (scipy.signal; Cellpose: Stringer et al., 2021; CASCADE: Rupprecht et al. 2021; CaImAn: Pnevmatikakis and Giovannucci, 2017; Napari: 10.5281/zenodo.3555620).

For manuscripts utilizing custom algorithms or software that are central to the research but not yet described in published literature, software must be made available to editors and reviewers. We strongly encourage code deposition in a community repository (e.g. GitHub). See the Nature Portfolio guidelines for submitting code & software for further information.

Data

Policy information about availability of data

All manuscripts must include a data availability statement. This statement should provide the following information, where applicable:

- Accession codes, unique identifiers, or web links for publicly available datasets
- A description of any restrictions on data availability
- For clinical datasets or third party data, please ensure that the statement adheres to our policy

Due to the large size, data generated and analysed in this study are available from the authors upon request. A source data file (Source Data.xlsx) is provided with this paper.

April 2023

Research involving human participants, their data, or biological material

Policy information about studies with human participants or human data. See also policy information about sex, gender (identity/presentation), and sexual orientation and race, ethnicity and racism.

Reporting on sex and gender	Not Applicable but stated in the Methods
Reporting on race, ethnicity, or other socially relevant groupings	Not Applicable
Population characteristics	Not Applicable
Recruitment	Not Applicable
Ethics oversight	Not Applicable

Note that full information on the approval of the study protocol must also be provided in the manuscript.

Field-specific reporting

Please select the one below that is the best fit for your research. If you are not sure, read the appropriate sections before making your selection.

☒ Life sciences ☐ Behavioural & social sciences ☐ Ecological, evolutionary & environmental sciences

For a reference copy of the document with all sections, see nature.com/documents/nr-reporting-summary-flat.pdf

Life sciences study design

All studies must disclose on these points even when the disclosure is negative.

Sample size	No statistical methods were used to define sample size, which was defined based on previous published similar work from our laboratory.
Data exclusions	For the analysis of frequency (waves or calcium spikes) only recordings of at least 5 minutes were considered. This is stated in several places within the Methods.
Replication	Animals were taken from multiple cages and breeding pairs.
Randomization	Animals of either sex were randomly assigned to the different experimental groups.
Blinding	No applicable - we only use mice expressing a specific fluorescent reporter.

Reporting for specific materials, systems and methods

We require information from authors about some types of materials, experimental systems and methods used in many studies. Here, indicate whether each material, system or method listed is relevant to your study. If you are not sure if a list item applies to your research, read the appropriate section before selecting a response.

Materials & experimental systems

n/a	Involved in the study	
☒	☐	Antibodies
☒	☐	Eukaryotic cell lines
☒	☐	Palaeontology and archaeology
☐	☒	Animals and other organisms
☒	☐	Clinical data
☒	☐	Dual use research of concern
☒	☐	Plants

Methods

n/a	Involved in the study	
☒	☐	ChIP-seq
☒	☐	Flow cytometry
☒	☐	MRI-based neuroimaging

Animals and other research organisms

Policy information about studies involving animals; ARRIVE guidelines recommended for reporting animal research, and Sex and Gender in Research

Laboratory animals	GCaMP6ffl/fl (Jackson Lab., stock number 028865); Atoh1-Cre (Jackson Lab., stock number 011104); Snap25-GCaMP6s (Jackson Lab., stock number 025111) ; NeuroD1-Cre (donated by Dr. Pavlínková: Li et al., 2012); Myo15-Cre (donated by Dr Safieddine: Caberlotto et al., 2011); Pax2-Cre (donated by Dr. Grove: Ohyama and Groves, 2004). CaV1.3-/- (donated by Dr Striessnig, Platzer et al. 2000); Otof-/- (donated by Drs Petit and Safieddine, Roux et al.2006).
Wild animals	Not Applicable
Reporting on sex	Animals of either sex were use. Sex was not considered in this study.
Field-collected samples	Not Applicable
Ethics oversight	The animal work was licensed by the UK Home Office under the Animals (Scientific Procedures) Act 1986 (PPL_PCC8E5E93) and was approved by the University of Sheffield Ethical Review Committee (180626_Mar).

Note that full information on the approval of the study protocol must also be provided in the manuscript.

Plants

Seed stocks	Not Applicable
Novel plant genotypes	Not Applicable
Authentication	Not Applicable
